# A coronary event without coronary artery disease: the hidden role of inherited thrombophilia—a case report

**DOI:** 10.1093/ehjcr/ytag714

**Published:** 2026-09-19

**Authors:** Nika Kuridze, Revaz Sturua, Levan Tsaguria

**Affiliations:** Caucasus Medicine School, Caucasus University, 1 Paata Saakadze st. 0102, Tbilisi, Georgia; Inpatient Unit of Cardiac Surgery Department, G. Chapidze Emergency Cardiology Center, 1 N. Javakhishvili st. 0159, Tbilisi, Georgia; Inpatient Unit of Cardiac Surgery Department, G. Chapidze Emergency Cardiology Center, 1 N. Javakhishvili st. 0159, Tbilisi, Georgia; Inpatient Unit of Cardiac Surgery Department, G. Chapidze Emergency Cardiology Center, 1 N. Javakhishvili st. 0159, Tbilisi, Georgia

**Keywords:** Coronary artery thrombosis, Inherited thrombophilia, ST-segment elevation myocardial infarction, case report

## Abstract

**Background:**

ST-segment elevation myocardial infarction (STEMI) in young patients without traditional cardiovascular risk factors requires evaluation beyond atherosclerotic disease. Inherited thrombophilia and cancer-related prothrombotic states represent important alternative aetiologies.

**Case summary:**

A 42-year-old man presented with an acute inferior STEMI. Coronary angiography demonstrated thrombotic occlusion of the proximal right coronary artery, treated with drug-eluting stent implantation. The patient had no cardiovascular risk factors. His medical history was significant for seminomatous testicular cancer treated with orchiectomy and cisplatin-based chemotherapy 10 months prior. Laboratory and imaging findings did not support atherosclerotic disease, and tumour markers were within normal limits. Given the absence of conventional risk factors, further evaluation was undertaken. Genetic testing revealed inherited thrombophilia. Following multidisciplinary consultation, antithrombotic therapy was optimized by adding rivaroxaban and adjusting antiplatelet therapy. At 1-year follow-up, the patient remained asymptomatic with no recurrent thrombotic events.

**Discussion:**

This case highlights the importance of a systematic and personalized diagnostic approach in young STEMI patients without evident coronary artery disease. Although inherited thrombophilia was considered a plausible underlying cause of coronary thrombosis in this case, the coexistence of prior malignancy and cisplatin-based chemotherapy may have further amplified the patient’s prothrombotic state. Therefore, it is probable that multiple pathogenic mechanisms coexisted and contributed to the development of coronary thrombosis. While typically linked to venous thromboembolism, inherited thrombophilia may also contribute to arterial thrombosis in selected patients, with important implications for long-term management.

Learning points

- STEMI in young patients without traditional cardiovascular risk factors warrants a systematic aetiological workup and multidisciplinary management.
- Inherited thrombophilia, though uncommon and frequently underdiagnosed, should be considered as a potential cause of coronary arterial thrombosis.
- Management of coronary thrombosis in inherited thrombophilia includes standard therapy with individualized consideration of adjunctive anticoagulation.

## Introduction

Global analyses indicate that cardiovascular diseases and cancer remain the leading causes of mortality worldwide.^1^ Due to the complex and multifactorial etiopathogenesis of coronary artery thrombosis, establishing an accurate diagnosis remains a significant clinical challenge, particularly in patients with malignancy who are undergoing or have previously received chemotherapy.^2^

Acute coronary syndrome occurring at a young age is uncommon and is frequently not associated with traditional cardiovascular risk factors, necessitating an expanded, systematic, and personalized diagnostic approach.^3^

In cases of myocardial infarction due to coronary thrombosis in the absence of evident obstructive coronary atherosclerosis, a thorough evaluation for non-traditional prothrombotic factors is warranted, including inherited thrombophilias, malignancy, and chemotherapy-associated prothrombotic states.

This case describes a young patient who developed ST-segment elevation myocardial infarction (STEMI) in the absence of conventional cardiovascular risk factors, in the context of a prior history of seminomatous testicular cancer (seminoma) treated with cisplatin-based chemotherapy and genetically confirmed inherited thrombophilia. The case highlights the importance of targeted differential diagnosis, multidisciplinary clinical decision-making, and individualized antithrombotic therapy in the management of coronary thrombosis in young patients.

## Summary figure

Timeline of clinical course

**Figure:**
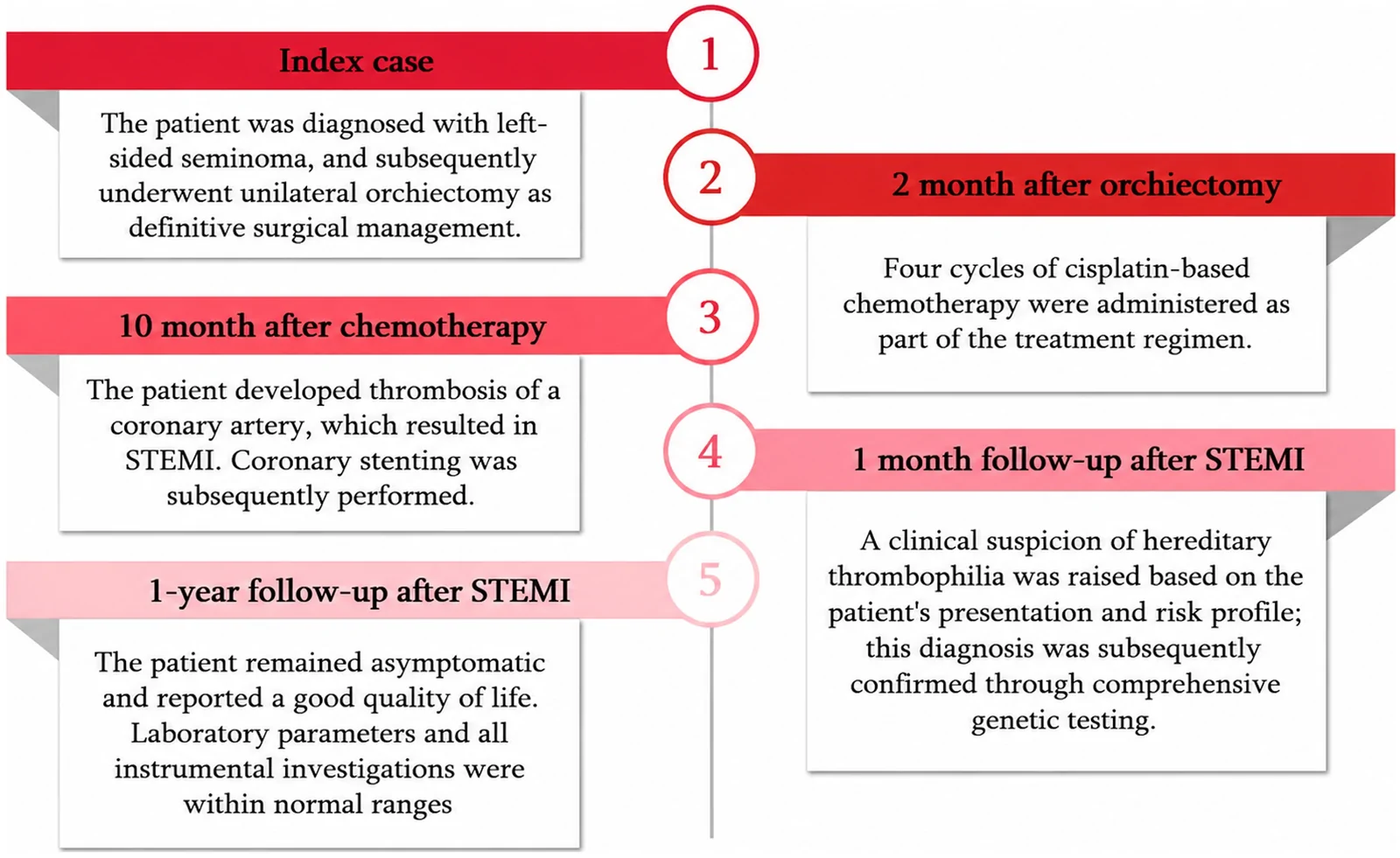


## Case presentation

A 42-year-old Caucasian man was referred for planned outpatient cardiology evaluation 1 month after an acute STEMI. At the index event, he presented hours after symptom onset. Electrocardiography demonstrated ST-segment elevation in the inferior leads (II, III, and aVF) (*Figure 1*). Transthoracic echocardiography demonstrated a preserved left ventricular ejection fraction of 56%, with normal right ventricular size and systolic function (tricuspid annular plane systolic excursion -20 mm), and no echocardiographic evidence of right ventricular involvement. Emergent coronary angiography revealed a thrombotic occlusion [thrombolysis in myocardial infarction (TIMI) thrombus grade 4] of the proximal right coronary artery (RCA) in the absence of significant underlying atherosclerotic stenosis (*Figure 2A*; Supplementary material online, *Video S1*). The remaining coronary arteries (left main, left anterior descending, and left circumflex) were with no evidence of atherosclerotic plaque or thrombotic lesions. The RCA was successfully treated with implantation of a single drug-eluting stent (*Figure 2B*; Supplementary material online, *Video S2*).

**Figure 1:**
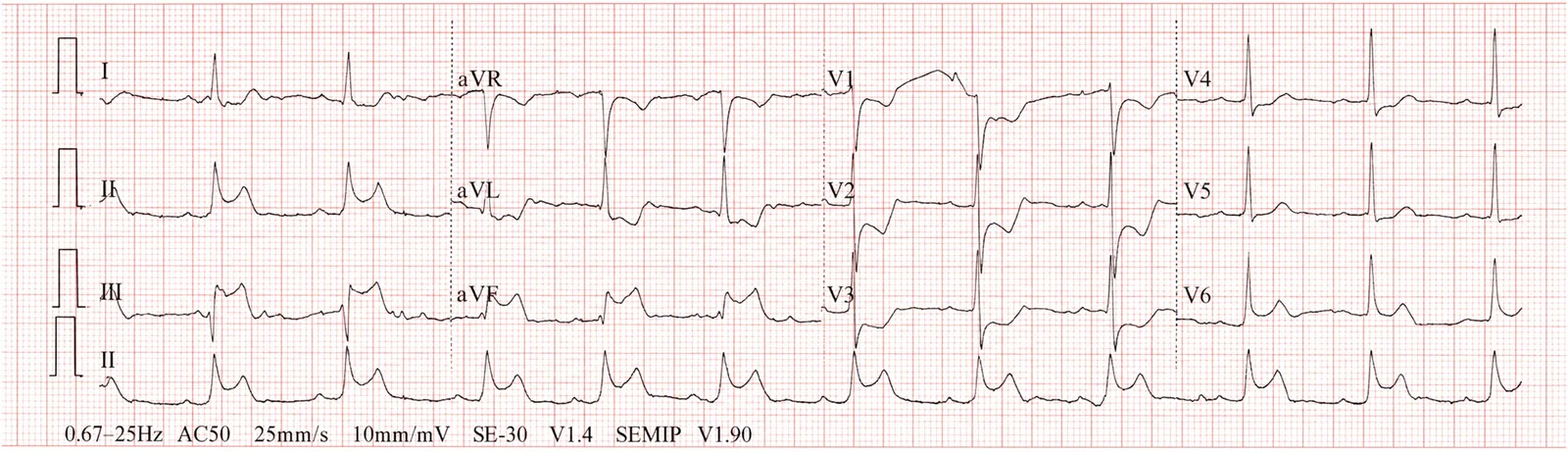
Admission ECG demonstrating inferior ST-segment elevation.

**Figure 2:**
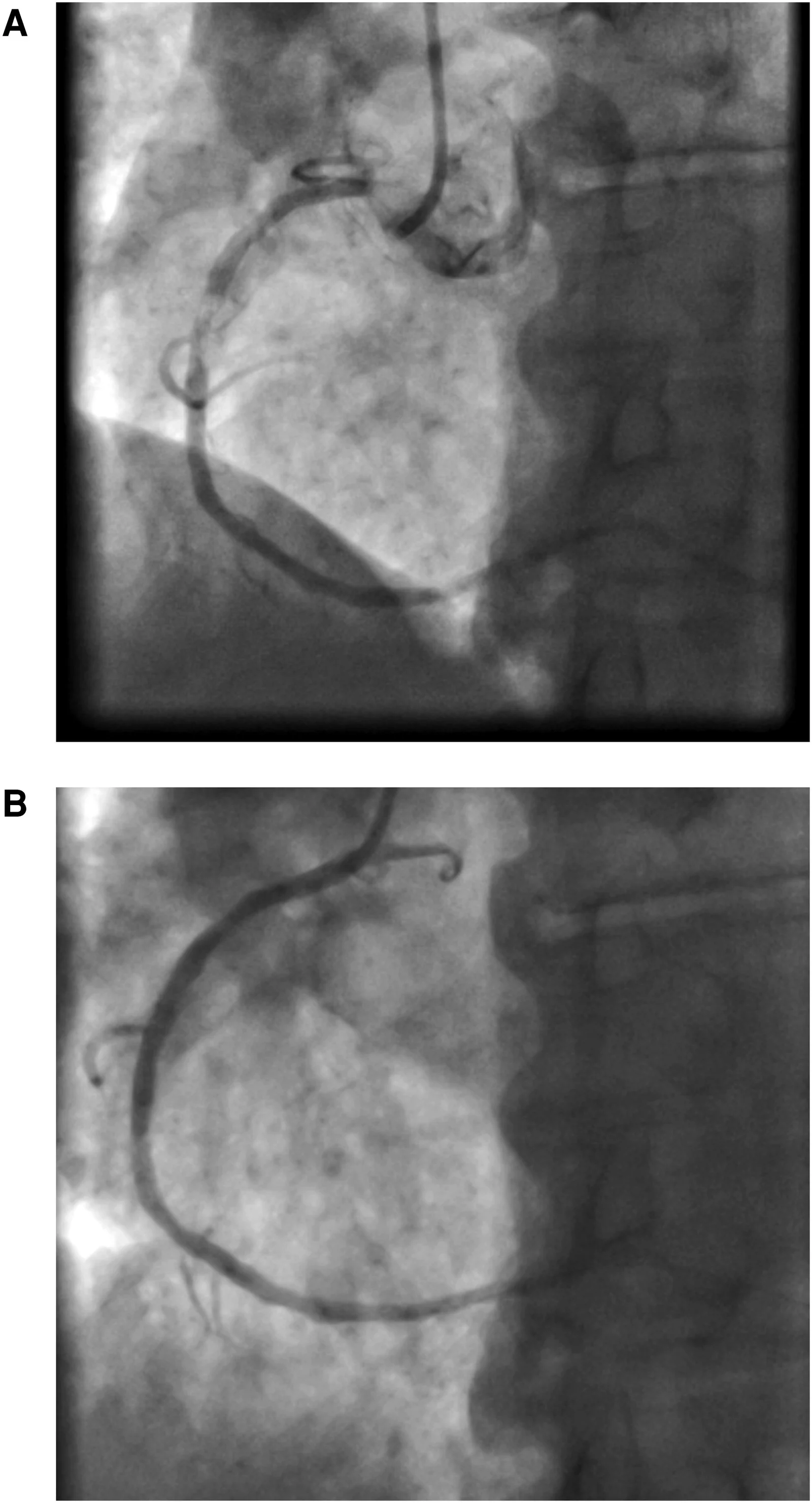
Coronary angiography (*A*) and percutaneous coronary intervention (*B*) of the right coronary artery.

The patient was hospitalized for 72 h and discharged on dual antiplatelet therapy (aspirin 100 mg, ticagrelor 90 mg b.i.d.), atorvastatin 40 mg, nebivolol 5 mg, and perindopril 5 mg, with cardiology follow-up at 1 month.

At follow-up, examination was unremarkable: BMI 24 kg/m^2^, BP 120/70 mmHg, HR 67 bpm. ECG showed sinus rhythm with T-wave inversion in lead III (*Figure 3*). LDL was 55 mg/dL. Other routine laboratory tests and transthoracic echocardiography revealed no abnormalities.

**Figure 3:**
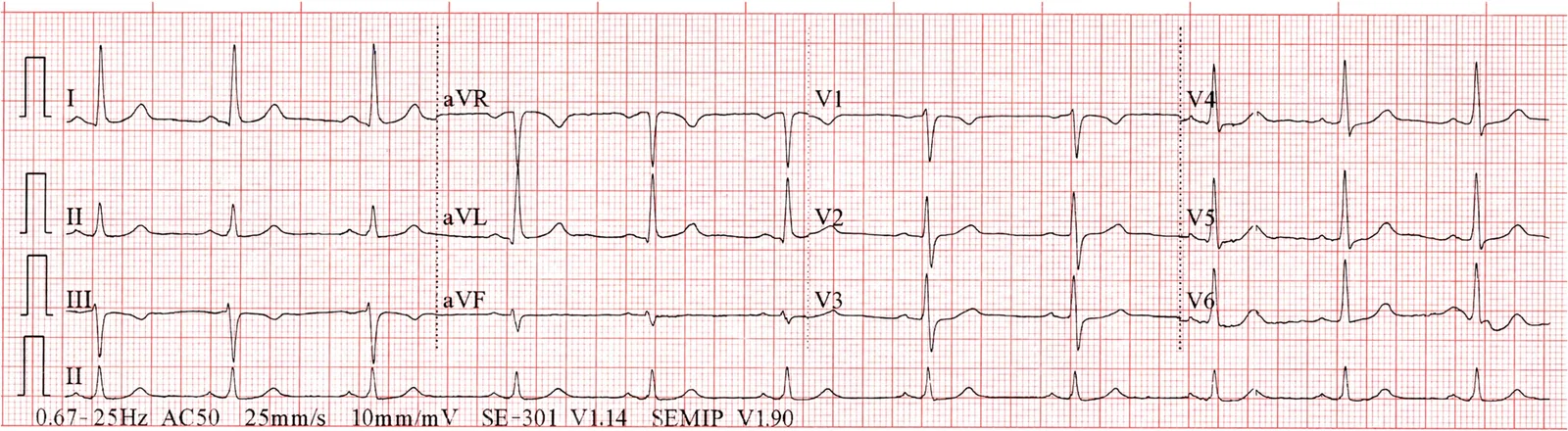
ECG at 1-month follow-up.

The patient had no prior history of cardiovascular disease or traditional cardiovascular risk factors, and the family history was negative. Notably, his history included an early-stage left testicular seminomatous germ cell tumour diagnosed 1 year earlier, treated with unilateral orchiectomy and four cycles of cisplatin-based chemotherapy, completed ∼10 months before the myocardial infarction.

Given the patient’s young age, prior regular physical activity, absence of tobacco use, and only occasional alcohol consumption, further evaluation for familial dyslipidemia was undertaken. Genetic testing for familial hypercholesterolaemia (LDLR, PCSK9, APOB) was negative, and Lp(a) levels were within the normal range.

Given the patient’s history of testicular cancer and exposure to chemotherapy, a malignancy- and treatment-related prothrombotic state initially appeared to provide a plausible explanation for the coronary thrombosis. However, despite the apparent temporal and biological association between prior cisplatin-based chemotherapy and the thrombotic event, a broader differential diagnosis was pursued, including inherited thrombophilia.

Accordingly, comprehensive testing for hereditary thrombophilia was performed. Genetic analysis identified a homozygous Factor V Leiden mutation together with a heterozygous Prothrombin G20210A mutation, confirming inherited thrombophilia. Following multidisciplinary consultation with a haematologist, and considering the underlying diagnosis and thrombotic risk, anticoagulation with a direct oral anticoagulant was initiated. Dual antithrombotic therapy with rivaroxaban and clopidogrel was prescribed—rivaroxaban 20 mg od. was added, ticagrelor was replaced with clopidogrel 75 mg od., and aspirin was discontinued. The patient was scheduled for routine follow-up in accordance with standard cardiovascular management recommendations.

At 1-year follow-up after STEMI, the patient remained asymptomatic and reported a good quality of life. Laboratory parameters and all instrumental investigations were within normal ranges, and the recommended LDL-cholesterol target was maintained. Antithrombotic therapy was simplified to rivaroxaban 20 mg od. as monotherapy. Additionally, atorvastatin 40 mg and perindopril 5 mg were continued as part of the patient’s long-term medical therapy. Continued clinical follow-up with periodic reassessment of ischaemic risk was advised.

## Discussion

Cancer itself, as well as antineoplastic therapies, especially platinum-based agents, are associated with endothelial dysfunction, platelet activation, and a sustained prothrombotic state, thereby increasing the risk of both venous and arterial thrombotic events.^4^

Following comprehensive instrumental and laboratory investigations, along with a detailed analysis of the patient’s medical history, several potential aetiologies for the development of coronary thrombosis were considered. The first and most common cause evaluated was type 1 myocardial infarction associated with ischaemic heart disease, resulting from thrombosis secondary to rupture of an atherosclerotic plaque.^5^ However, diagnostic workup, including the lipid profile, familial hypercholesterolaemia, LP(a), did not reveal evidence supporting an underlying atherosclerotic aetiology. The ESC Guidelines recommend intracoronary imaging [optical coherence tomography (OCT) and intravascular ultrasound (IVUS)] during angiography to differentiate underlying mechanisms, such as plaque disruption or spontaneous coronary artery dissection. Although intracoronary imaging was unavailable, the presence of an overt thrombotic occlusion combined with the absence of angiographic atherosclerosis directed our focus towards the evaluation of systemic prothrombotic states.

An alternative hypothesis was tumour-associated thrombosis, which has been reported as a potential mechanism of thrombotic events in oncological patients.^6^ Evaluation of this possibility was challenging, as the malignancy was diagnosed at an early stage and managed with timely surgical intervention. Postoperatively, tumour markers including human chorionic gonadotropin, alpha-fetoprotein, and lactate dehydrogenase were within normal limits, and the tumour was classified as stage I (SGCT-I). In addition, four cycles of adjuvant chemotherapy were administered to reduce the risk of recurrence and to avoid further complications. These findings made tumour-induced thrombosis an unlikely explanation and supported the conclusion that coronary artery thrombosis in this patient was more likely attributable to an alternative pathological mechanism.

The third hypothesis considered was cisplatin-induced thrombosis, a well-documented complication in oncology patients receiving platinum-based chemotherapy.^7^ However, according to a large retrospective study, vascular thrombosis occurred predominantly within the first 100 days after initiation of cisplatin, while arterial thrombosis was observed in a relatively small proportion of cases, ∼8.3%.^8^ Consistently, the EAU guideline on testicular cancer recognizes the increased risk of thromboembolic events, predominantly venous, associated with cisplatin-based chemotherapy and recommends thromboprophylaxis in high-risk patients but does not specifically address arterial thrombosis or myocardial infarction.^9^ Given the predominance of venous over arterial events in cisplatin-associated thrombosis and the typical peak risk within 3–4 months of chemotherapy, the 10-month interval in this case makes cisplatin-induced thrombosis an unlikely cause of coronary thrombosis.

During the evaluation of potential causes of arterial thrombosis, differential diagnosis with inherited thrombophilia was considered essential. Based on a comprehensive review of the patient’s clinical history and laboratory findings, an inherited prothrombotic disorder was suspected. Genetic testing was therefore performed and confirmed the diagnosis of hereditary thrombophilia, providing a plausible aetiological explanation for the development of coronary artery thrombosis in this patient. Furthermore, it is biologically plausible that persistent cisplatin-induced endothelial dysfunction and platelet activation acted synergistically with the underlying inherited thrombophilic state, together contributing to the development of coronary thrombosis.

Although inherited thrombophilia is a well-established risk factor for venous thromboembolism, its role in arterial thrombosis is limited.^10,11^ However, it has been recognized as a significant risk factor for coronary artery disease in specific subgroups, particularly in young individuals.^12–14^ The patient’s pre-existing prothrombotic state, together with the presence of multiple inherited thrombophilic variants, may have created a cumulative prothrombotic milieu, predisposing to intracoronary thrombus formation leading to STEMI. In addition, arterial thrombus formation may have been facilitated by transient triggers, such as endothelial injury or dysfunction and local vascular inflammation.

### Limitations

A limitation of this case is that intracoronary imaging (OCT/IVUS) could not be performed due to its unavailability at our institution.

## Conclusion

This case highlights the complexity of thrombotic risk assessment in young patients with STEMI and underscores the importance of considering inherited thrombophilia when conventional cardiovascular and treatment-related causes are excluded. A systematic, stepwise aetiological evaluation allowed identification of a plausible underlying prothrombotic condition, emphasizing the need for individualized diagnostic strategies to guide optimal management in inherited thrombophilia-associated arterial thrombosis.

## Lead author biography


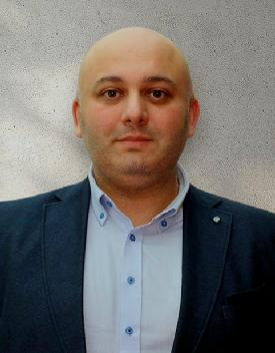
Dr Nika Kuridze, MD, PhD, FESC, is a cardiologist and cardiac electrophysiologist based in Tbilisi, Georgia. He serves as the Head of the Inpatient Unit of Cardiac Surgery at G. Chapidze Emergency Cardiology Center. His research focuses on the impact of cardiac implantable electronic devices on patients’ quality of life. He is also affiliated with the Caucasus Medicine School of Caucasus University, where he contributes to medical education and academic activities.

## Supplementary Material

ytag714_Supplementary_Data

## Data Availability

All available data are presented in the article and in its online Supplementary material.
